# Food Sovereignty: Power, Gender, and the Right to Food

**DOI:** 10.1371/journal.pmed.1001223

**Published:** 2012-06-26

**Authors:** Rajeev C. Patel

**Affiliations:** School of Development Studies, University of KwaZulu-Natal, Durban, KwaZulu-Natal, South Africa

## Abstract

In an article that forms part of the *PLoS Medicine* series on Big Food, Raj Patel examines the concept of food sovereignty, which aims to address inequalities in power that characterize the global food system and fuel hunger and malnutrition.

**Summary Points**

- Understanding hunger and malnutrition requires an examination of what systems and institutions hold power over food.
- The concept of “food security” captures the notion of hunger not as a deficit of calories, but as a violation of a broader set of social, economic, and physical conditions.
- Gender is key to food insecurity and malnourishment, because women and girls are disproportionately disempowered through current processes and politics of food's production, consumption, and distribution.
- La Via Campesina has advocated for food sovereignty, through which communities have the right to define their own food and agriculture policy. Women's rights are central elements to food sovereignty.
- The role of the food industry demands attention within the food system, where power is concentrated in the hands of a few corporations.

*This article was commissioned for the* PLoS Medicine *series on Big Food that examines the activities and influence of the food and beverage industry in the health arena.*

## Power over Food

One of the most enduring misconceptions about hunger is that it is primarily the result of a deficit in global food production. If this were so, we might expect food to be absent at times and in places where people die of hunger. Yet economist Amartya Sen has shown that in the majority of cases of widespread famine-related death since WWII, food has been available within the famine-affected area. People have died not for want of food, but for want of the entitlement to eat it [1]. Questions about hunger and its attendant pathologies, therefore, ought to begin with questions about social and political configurations around *power* over food, rather than about the mere presence or absence of food in the vicinity of a hungry individual.

Although no single commonly agreed definition of hunger exists, two common standards prevail: “undernourishment” and “food security.” The former refers to the number of people “whose dietary energy consumption is continuously below a minimum dietary energy requirement for maintaining a healthy life and carrying out a light physical activity” [2]. Undernourishment is a condition suffered by individuals. It is, however, usually established not through individual surveys but through an analysis of a country's food availability, household purchasing power, and entitlements [3],[4]. Current estimates put the worldwide number of undernourished people at nearly one billion [3].

The concept of “food security” attempts to capture the notion of hunger as a deficit not of calories, but as a violation of a broader set of social, economic, and physical conditions. In 1996, the Food and Agriculture Organization of the United Nations (FAO) established at its World Food Summit the most widely agreed definition [5] that “Food security, at the individual, household, national, regional and global levels [is achieved] when all people, at all times, have physical and economic access to sufficient, safe and nutritious food to meet their dietary needs and food preferences for an active and healthy life.”

By definition, more people are food insecure than are undernourished, and food insecurity precedes undernourishment. Although there are few people in the United States whose calorie intake is continuously below the threshold of a maintaining healthy life, there are many who, at some point during any given year, are unable to meet their food needs. According to the United States Department of Agriculture (USDA), in 2010 there were 48.8 million US citizens living in food-insecure households. The distribution of food insecurity is uneven. In the US, 21.6 million children lived in food-insecure households, and 35.1% of all female-headed households were food insecure in 2010, compared to 25.4% of male-headed households [6].

Since food insecurity is a broader measure than that of undernourishment, it has been correlated both with hunger and obesity, particularly among women [7]. If hunger is a symptom of a lack of control over the socioeconomic context in which one attempts to eat, it is not unreasonable to understand that lack of control as correlated with factors associated with obesity too. It is possible to have sufficient calories, but insufficiently nutritious food for a healthy life. Armed with this understanding, and with persistent evidence across countries of women and girls' disempowerment compared to men and boys [8], it becomes easier to appreciate the systematically higher rates of food insecurity among women.

## Gender and Food

The link between gender and food becomes clearer through an understanding of power and control in the food system. Giving away food does little to address the underlying causes of disempowerment that lead to hunger [9]. One group that has articulated this is an international peasant movement called La Via Campesina (see Box 1). They argue that if governments aim merely for food security as a policy goal, the politically difficult questions of inequality in power that produced food insecurity would be ignored, and a broken system would be patched up with entitlements [1]. It is possible, after all, to be food secure in prison where one might continually *access* safe and nutritious food, yet remain fundamentally disempowered over the process and politics of the food's production, consumption, and distribution.

**Box 1. La Via Campesina**

La Via Campesina is an organization of farmers, peasants, and landless workers' movements with over 150 million combined members in 70 countries [46]. Its first meeting was held in 1993, and it was constituted as an umbrella organization for a range of social movements that had, through the 1980s, begun to work more closely in Asia, the Americas, and Europe. These movements had come into contact with one another through their attempts to understand, resist, and offer alternatives to “free market” agricultural trade. Even before the organization was officially created, La Via Campesina's member organizations had undertaken a range of actions to confront what they saw as inequality in power within the food system. In India, 200,000 farmers protested the patenting of seeds by multinational corporations. In Europe, 30,000 farmers marched on Brussels to offer an alternative policy goal to the achievement of food security. In Brazil, hundreds of thousands of people occupied farmland, upon which they built thriving communities. In 1996, at the same World Food summit at which the most recent definition of food security was written, La Via Campesina codified its vision for an alternative food system under the rubric of “food sovereignty.” At a 2009 La Via Campesina meeting, one of the slogans offered by the assembly was that “food sovereignty is an end to all forms of violence against women.”

Instead of food security, Via Campesina has advocated for “food sovereignty.” Just like the definition of food security, food sovereignty is an evolving and many-faceted term, but it has an invariant core: “communities have the right to define their own food and agriculture policy” [10]. To be clear, sovereignty is not a call for self-sufficiency, for states to grow within their borders sufficient food to feed their citizens. La Via Campesina instead calls for people to be sovereign over their food systems, for people to have the power to decide what the system should look like. This is an intentionally vague call, with many questions left open-ended, so that the communities involved in claiming food sovereignty might answer issues around production, distribution, and consumption of food for themselves. It is through food sovereignty, La Via Campesina argues, that food security might be achieved, and undernourishment eradicated.

The main demand in food sovereignty is that, for the first time, decisions about the shape of the food system ought to be in the hands not of powerful corporations or geopolitically dominant governments [11], but up to the people who depend on the food system. For the discussion to be representative of the community's desires, however, a non-negotiable element of food sovereignty is women's rights. In order for a democratic conversation about food and agriculture policy to happen, women need to be able to participate in the discussion as freely as men.

Peasant movements, and those who support them, have been castigated as romantics pining for an unattainable past [12]. An insistence of women's rights places food sovereignty firmly in the twenty-first century. This has a practical purpose. Of those undernourished, 60% are women or girls [13]. It is hard to conceive a discussion about hunger without connecting the epidemiology of hunger to women's disempowerment.

On the production side of the food system, women constitute 43% of the agricultural workforce, more often involved in producing food for domestic consumption than export. They are discriminated against in issues ranging from land tenure to wages, from government support to access to technology. The FAO notes that “if women had the same access to productive resources as men, they could increase yields on their farms by 20–30 percent. This could raise total agricultural output in developing countries by 2.5–4 percent, which could in turn reduce the number of hungry people in the world by 12–17 percent” [14].

In addition, women stand to bear a disproportionate burden of the consequences of the twenty-first century's predicted global increase in non-communicable disease (NCD) prevalence. In South Asia, for example, NCDs are projected to account for 72% of deaths by 2030, up from 51% in 2008. In Sub-Saharan Africa, the estimates are 46%, up from 28% over the same period [15]. In addition to the duties of paid work, women bear a disproportionate burden of care work in the management of morbidity associated with NCDs [16],[17], especially in contexts of poverty [18]. These are the kinds of inequities to which food sovereignty calls attention.

## Systemic Inequity and the Right to Food

Beyond an examination of the inequitable distribution of power at a household level, food sovereignty suggests an investigation of power relations at meso- and macroeconomic levels. La Via Campesina members are, for example, concerned about corporate power within the global economy. The food system's dysfunction continues to be lucrative for a range of food and agriculture companies. Profits often derive from the increased consumption of processed food, which in turn have driven a global obesity epidemic. Yet the distribution mechanisms within the food system that ration food on the basis of ability to pay have produced the paradox of a billion hungry during a time when there are more than 1.5 billion people overweight [19],[20].

Within the food system, power is concentrated in the hands of a few corporations. In 2008, the top ten agrochemical corporations controlled almost 90% of the global sales of pesticides. Of the US$22 billion global proprietary seed market, only ten corporations controlled 67% [21]. In 2005, the top four beef packing firms controlled 83.5% of the market in the US [22], and worldwide, 40% of all groceries were sold by only 100 retailers [21]. These trends across the food industry have been on an almost-steady climb since they were recorded first in the 1970s. As the US government recently found, “for example, in the pork sector, the market share of the largest four hog slaughtering firms increased from 36 percent in 1982 to 63 percent in 2006. In addition, at the retail level, the share of grocery store sales held by the largest four firms more than doubled, from 16 percent in 1982 to 36 percent in 2005” [23].

This concentration of power has gendered consequences. In contexts where women perform the majority of horticultural and agronomic innovation, they can find their agroecological knowledge supplanted by the technologies of industrial agriculture. Pesticide companies own the largest seed companies, and their agricultural model, dependent on purchased supplies of hybrid seeds and chemical inputs, favors larger, more capital-intensive farms. Women have systematically less access to both land and capital than men, and despite an often sophisticated level of knowledge about farming systems, women's views seldom matter in the shaping of choices around agricultural technologies and food policy [24]. In addition, employment within agriculture consistently pays women around 25% less than men. When food is accessed through market mechanisms, this increases women's systemic risk of hunger [25].

It is for these reasons that women leaders within peasant movements have taken strong stands against multinational corporations such as Monsanto and Cargill [26]. To be sure, concentration of agricultural power is not new. At the turn of the nineteenth century, four firms—Dreyfus, Cargill, Continental, and Bunge—dominated global grain trading [27]. Today, however, the extent to which food markets matter is far greater. Agricultural market concentration is evident not only in international trade, but across domestic production, distribution, and consumption. This concentration matters more when there are fewer alternatives to the markets in which concentration occurs.

## The Role of Markets and Governments

To understand why the private sector has achieved such power, it is worth looking at other actors' roles within the food system. Philanthropic foundations have, for example, been responsible for advancing the kinds of industrial agriculture that has imperiled La Via Campesina's members [28],[29]. The “Green Revolution,” in which farmers were encouraged and sometimes forced by governments to adopt a system of farming involving hybrid seeds, fertilizer, and pesticides, was initially funded by the Rockefeller and Ford Foundations, and is currently being encouraged by the Gates Foundation in Africa [30],[31],[32]. These farming systems have had gender-negative impacts, as women's knowledge is excluded, and women are systematically less able to control the capital required to participate in resource-intensive farming [33],[34],[35].

National governments and international organizations have also been faulted for their behavior in shaping the food system. Of particular interest to La Via Campesina is the extent to which, through international economic agreements such as the World Trade Organization's (WTO's) Agreement on Agriculture, governments have enabled private sector markets to expand their influence within the food system. A central demand in La Via Campesina's call for food sovereignty is for the WTO to “get out of agriculture” [36]. By this they mean not only ought the Agreement on Agriculture within the WTO be nullified, but that a range of other WTO provisions that affect agriculture, such as rules on intellectual property rights on seeds and phytosanitary measures, also be suspended. Trade agreements rules are influenced by the corporations that subsequently benefit from them [37], with demonstrated gendered impacts as a result [38],[39].

Food corporations continue to attempt to shape domestic and international public policy. PepsiCo, for instance, has gone to great lengths to claim a place at the table in addressing public health issues [40]. Yet the company has since 2000 spent US$26.88 million on lobbying in the US [41], in particular in response to taxes on its products and voicing its concerns on restrictions on marketing its foods to children [42],[43]. PepsiCo's behavior is emblematic of a wider trend in private sector spending within the food system. In a context of shrinking public budgets, and the transformation of public institutions such as schools into sites for the sale of obesogenic products [44], the influence of private interest in public policy matters immensely. Yet the food industry is pushing public debate toward an interpretation of the rise of NCDs as fundamentally a problem of individuals [45]. To accept this is to urge a policy response in which NCDs can be remedied by better individual behavior, rather than more regulation. With women more responsible than men for children's diets, this has the effect of pathologizing individual women, rather than finding fault with a system that removes their freedom to make their children's diets healthier.

## Conclusion

The inequalities in power that characterize the food system can be found in households, corporations, regional and state governments, private philanthropic foundations, and international organizations. The strengths of a food sovereignty approach lie in the heuristic approach to power relations that it invites, particularly with respect to gender. For La Via Campesina, and many others, identifying inequities in power within the global food system is more than an academic exercise—it is a means not only to interpret the system, but also to change it.

## Abbreviations

FAO: Food and Agriculture Organization of the United Nations
NCD: non-communicable disease
USDA: United States Department of Agriculture
WTO: World Trade Organization
